# Natural Resources of Medicinal and Cosmetic Plants

**DOI:** 10.3390/plants11091251

**Published:** 2022-05-05

**Authors:** Adam Stebel

**Affiliations:** Department of Pharmaceutical Botany, Faculty of Pharmaceutical Sciences in Sosnowiec, Medical University of Silesia in Katowice, Ostrogórska 30, 41-200 Sosnowiec, Poland; astebel@sum.edu.pl

This Special Issue (SI) of *Plants* is devoted to medicinal and cosmetic plants. In some regions of the world, they are still the basis for the production of medicines and cosmetics. For many years, in highly developed countries, an increase in interest in medicinal and cosmetic products based on raw plant materials has been observed.

The proposed topics in the current SI included five areas: (1) natural plant resources in particular regions and countries, (2) plant protection, (3) searching for new medicinal and cosmetic species or their properties, (4) ethnobotany as a source of information about medicinal and cosmetic plants, and (5) human impact on the environment and medicinal and cosmetic plants.

Finally, 13 papers were collected for the SI, including 11 original research papers and 2 reviews. The vast majority of them correspond to topics related to the third area. Some of these papers were inspired by information on the use of plants in traditional medicine, such as *Artemisia argyi* H. Lév. & Vaniot, *Houttuynia cordata* Thunb., *Carthamus tinctorius* L., and *Thunbergia laurifolia* Lindl. Interesting is the group of articles on the antimicrobial properties of extracts from plants, for example, *Limonium binervosum* (G. E. Sm.) C. E. Salmon and *Silybum marianum* L., which can be used in the control of phytopathogens in ecological plant crops.

The authors in their works undertook to solve the following problems:

*Carthamus tinctorius* has been used in traditional treatment for cardiovascular, cerebrovascular, and diabetic vascular complications. Lee et al. [1] investigated how the ethanol extract of *C. tinctorius* can be used ethnopharmacologically and alleviate vascular inflammatory processes under cytokine stimulation in human vascular endothelial cells. They suggest the potential role of ECT as a beneficial therapeutic herb in vascular inflammation via the ROS/NF-kB pathway, and the regulation of the Nrf-2/HO-1 signaling axis is involved in its vascular protection.

*Houttuynia cordata* is a native herbaceous plant, possessing many biological properties including antioxidant and anti-inflammatory activities, used in traditional medicine in some Asian countries. Mapoung et al. [2] showed that the hyperoside-enriched fraction of H. cordata exerted potent anti-skin aging properties against UVB exposure and can potentially be used in pharmaceutical or cosmetic formulations as a photoprotective or anti-skin aging agent.

The aim of the research by Jarco et al. [3] was to compare the free-radical-scavenging ability of the infusions of *Asparagus racemosus* Willd. root and herbs of *Mitchella repens* L., *Cnicus benedictus* L., *Galega officinalis* L., and *Eupatorium cannabinum* L. and to determine the influence of UVA irradiation of the plant materials on interactions of these infusions with free radicals. They showed that UVA radiation reduces the antioxidant interactions of all tested infusions, especially of *Eupatorium cannabinum*.

The antimicrobial activity of *Hibiscus syriacus* L. ‘Mathilde’ extracts has been investigated in vitro by Sánchez-Hernández et al. [4] against some bacteria that cause diseases of cultivated plants. The results shows that this species may be a promising source of antimicrobials for plant crop protection, including, e.g., the organic cultivation of medicinal plants.

*Limonium binervosum* is a halophytic plant, occurring on the Atlantic coasts of Western Europe. The work of Sánchez-Hernández et al. [5] for the first time provides its phytochemical constituents as well as information about the antimicrobial activity of its extracts against some phytopathogens.

Hyperuricemia, the abnormally excess accumulation of uric acid, is caused by an imbalance between the production and excretion of uric acid and is a major cause of gout. Kim et al. [6] compared the effects of treatment with extracts from *Chrysanthemum indicum* L. and *Cornus officinalis* Siebold & Zucc., both individually and in combination, on hypoxanthine-treated human liver cancer cells, primary mouse renal proximal tubule cells, and potassium-oxonate-induced hyperuricemic mice to develop agents for the prevention of hyperuricemia. The results suggest that a combination of these species may potentially be used for the development of effective natural anti-hyperuricemic functional foods.

*Thunbergia laurifolia* is a traditional medicinal plant in Southeast Asia. The results of investigations conducted by Pattananandecha et al. [7] showed that standardized rosmarinic-acid-enriched extract prepared from *T. laurifolia* leaves showed antioxidant activity and anti-photoaging effects, and it is a potential candidate as a natural active ingredient for cosmeceutical product application.

Mapoung et al. [8] presented the results of research on samples of commercial cosmetic creams on the Thai market of their antioxidant activity, tyrosinase inhibitory effects, and phenolic contents. They demonstrated that the total phenolic contents in the functional cosmetic creams could play a role in antioxidant activity and anti-tyrosinase activities.

*Silybum marianum* is widely known for its hepatoprotective properties. Langa-Lomba et al. [9] present a phytochemical analysis of the extracts of *S. marianum* capitula during the flowering phenological stage and the application of the hydro-methanolic extracts as an antifungal agent for plant crop protection.

*Artemisia argyi* is widely used in traditional medicine in East Asia. Shin et al. [10] analyzed its anti-inflammatory activity and molecular mechanisms using RAW264.7 cells line, then evaluated the curative efficacy in rats with acute gastric ulcers. *A. argyi* exhibited strong anti-inflammatory effects and contributed to the modulation of HCl-EtOH-induced gastric ulcer in rats.

*Glycyrrhiza* is a type of herbaceous perennial legume used in traditional herbal medicine in many countries. Li et al. [11] studied the genetic aspects of abiotic stresses and hormone therapies on the development and metabolism of secondary metabolites in selected species of this genus.

Torres-Contreras et al. [12] summarized the research in Mexican plants related to UV protection, presented the most studied Mexican plants and the photoprotective molecules found in them and analyzed the studies conducted to elucidate the mechanism of photoprotection of those molecules and their potential use as ingredients in sunscreen formulas.

Drobnik and Stebel [13] compiled historical information on the use of bryophytes in European medicine. They related these data to the modern knowledge of their phytochemistry.

To sum up, the papers included in this SI brought a lot of new information on the possibility of using several species of plants occurring in various regions of the world. We encourage researchers to read the articles.
